# Friends of Theological Seminaries

**Published:** 1854-04

**Authors:** 


### FRIENDS OF THEOLOGICAL SEMINARIES,

TENS of thousands of dollars are expended every year in sustaining the Seminaries which prepare young men for the Ministry, and tens of thousands besides in maintaining some of those young men who are not able to support themselves. It is well known that many a dollar thus expended has been earned by the manual labor of the pious and humble poor, whose pittance, small as it is, is bestowed at a sacrifice of personal convenience and comfort in some instances, and little children are taught to deny themselves some of the common eatables of life that the worth of them may be thrown in the treasury of the Church. *Is it right?*—IS IT RIGHT?—IS IT RIGHT? ye Secretaries of beneficiary treasuries, that this money shall be expended in fitting young men for the Ministry, when no pains are taken to maintain and secure that good health which is essential to their efficiency? Is one single lecture read to these young men, during the whole three years' course of theological study, which instructs them how to preserve health, the high duty of thus preserving it,—of the criminality of its neglect? Deacons, Elders, Vestrymen, Priests, Rectors, Bishops of the Church, think of it, simply think of it, and answer me—is 't wise?—Is it right? The most practicable method which suggests itself to my mind at this time, and perhaps the most economical, is the following:—

Let the friends of any particular Seminary send me the money necessary to pay for fifty, or a hundred, or more copies of the *Journal.* I will obtain an alphabetical list of all the Students in the Seminary designated, and if money enough is not sent to pay for a copy for each, the monthly numbers may be sent to them in rotation, and out of each valuable information may be obtained, which does not appear in books accessible to them. The one article in the February number on "*Air and Exercise*" cannot be carried out by any sedentary man, not an actual invalid, without giving him a degree of strength and vigor, bodily and mental, to which he has hitherto been a stranger.

I feel no hesitation in making these statements, because I know that the subject is an important one, that it has been long neglected, and by that neglect the Church suffers every year in the premature loss of many among its best men. I know that the evil can be remedied to a considerable extent, and no time is more suitable than the "NOW;" when the relative number of theological students in almost every branch of the Church is decreasing, and has been for several years, while the daily tide of foreign immigration is rolling in its hundreds, and sometimes its thousands, multitudes of whom are steeped in the foulest, darkest poison of the baldest infidelity. Let them come, but invite them to you by kindliness, and when you have thus made them love you as a brother, be prepared to win them over to the better way, by teachers who have steady heads and sound hearts, in sound, vigorous, and healthful bodies, for such are the men to preach the truth with a will, and with a power too, to which the poor, pale, wan, dyspeptic youth must for ever be a stranger. Can a dyspeptic man, or a chronic invalid be trusted to preach the truth always? I think not. The supposition is a contradiction, it is a flat absurdity. A sound mind in a sound body, is as true now, as in the davs of the Latins.

